# Germplasm Resources and Genetic Breeding of Huang-Qi (Astragali Radix): A Systematic Review

**DOI:** 10.3390/biology13080625

**Published:** 2024-08-16

**Authors:** Pengbin Dong, Lingjuan Wang, Yong Chen, Liyang Wang, Wei Liang, Hongyan Wang, Jiali Cheng, Yuan Chen, Fengxia Guo

**Affiliations:** 1 College of Agronomy, College of Life Science and Technology, State Key Laboratory of Aridland Crop Science, Gansu Agricultural University, Lanzhou 730070, China; dongpb@stumail.nwu.edu.cn (P.D.); w18856278265@163.com (L.W.); 18298363660@163.com (W.L.); why8852@163.com (H.W.); chengjl@st.gsau.edu.cn (J.C.); 2 Pingliang City Plant Protection Centre, Pingliang 743400, China; wlj950369@163.com; 3 Institute of Soil, Fertilizer and Agricultural Water saving, Xinjiang Academy of Agricultural Sciences, Urumqi 830000, China; chenyong@xaas.ac.cn

**Keywords:** Astragali radix, breeding strategy, germplasm resources, germplasm innovation, variety breeding

## Abstract

**Simple Summary:**

*Astragalus* species (Huang-Qi), belonging to Fabaceae, are widely spread in the arid to semiarid environments in northeast, north, and northwest China, and most of the varieties thereof have dual-use functions as medicine and food, with the potential to become high-quality health food. However, compared to that for other crop species, scientific research on Huang-Qi germplasm resources and genetic breeding is lagging behind. This review systematically introduces Huang-Qi to the present germplasm resources, genetic diversity, and genetic breeding, including wild species and cultivars, and summarizes the breeding strategy for the cultivars in these species and the results thereof. This review will provide breeders with new insights into breeding Huang-Qi.

**Abstract:**

Huang-Qi (Astragali radix) is one of the most widely used herbs in traditional Chinese medicine, derived from the dried roots of *Astragalus membranaceus* or *Astragalus membranaceus* var. *mongholicus*. To date, more than 200 compounds have been reported to be isolated and identified in Huang-Qi. However, information pertaining to Huang-Qi breeding is considerably fragmented, with fundamental gaps in knowledge, creating a bottleneck in effective breeding strategies. This review systematically introduces Huang-Qi germplasm resources, genetic diversity, and genetic breeding, including wild species and cultivars, and summarizes the breeding strategy for cultivars and the results thereof as well as recent progress in the functional characterization of the structural and regulatory genes related to horticultural traits. Perspectives about the resource protection and utilization, breeding, and industrialization of Huang-Qi in the future are also briefly discussed.

## 1. Introduction

*Astragalus* L. (Fabaceae) comprises approximately 2900 species globally, distributed across the Northern Hemisphere, South America, and Africa and sparsely in North America and Oceania, with more than 250 taxonomic sections [1,2]. In China, there are 401 species, 221 of which are endemic, and 59 sections (2 endemic), mainly concentrated in the Himalayas. *Astragalus* L. species or cultivars are annual or perennial herbs, subshrubs, or shrubs. They are popularly used in livestock feed, medicine, food, cosmetics, and fodder worldwide [3]. According to reports in the literature, there are 29 species of *Astragalus* L. in China that can be used as medicines and/or foods, such as *Astragalus chinensis* (the seeds are used as medicine to treat neurasthenia and diabetes), *Astragalus melilotoides* (the whole herb is used as medicine to treat rheumatic pain and numbness of the limbs), *Astragalus yunnanensis* (the root is used to treat bleeding from the mouth and nose, toothache, measles, and tonsillitis; also used to relieve cough and dysentery in Tibet), and *A*. *membranaceus* var. *mongholicus* and *A*. *membranaceus* have been widely used as superfood ingredients in traditional Chinese medicine and health products in recent years [4,5]. Huang-Qi, formerly known as Astragali radix and first documented in ‘‘Shennong Bencao Jing’’, is the dried root of the legume plant *A*. *membranaceus* var. *mongholicus* or *A*. *membranaceus* and has the effects of invigorating Qi, promoting Yang, solidifying surfaces, acting as an antiperspirant, promoting diuresis, and reducing swelling [6,7]. In addition, Huang-Qi contains saponins, polysaccharides, flavonoids, trace elements, amino acids, and other active ingredients; modern pharmacological research has shown that it has a variety of pharmacological activities, including enhancing and bidirectionally regulating the body’s immune functions; antiviral, anti-aging, anti-fatigue, anti-inflammatory, and analgesic functions; inhibiting tumors; and a variety of pharmacological activities [8,9].

In fact, China is the largest producer and exporter of Huang-Qi in the world [10]. In recent years, there has been a huge domestic demand for Huang-Qi, with approximately 50% of it being used for the production of Chinese herbal medicine pieces and the other nearly 50% used for making traditional Chinese medicines and extracts and other formulations [11]. In addition, in the international herbal medicine market, the production and exportation of Huang-Qi are exclusive to China, having once led among the seven categories of Chinese medicinal herbs exported, with values exceeding USD 10 million [12]. Intense demand has led to the near-exhaustion of wild Huang-Qi resources. Initially, Huang-Qi was harvested from the wild, with the main production regions including Shanxi, Inner Mongolia, Gansu, Hebei, and Ningxia [13]. However, with the increase in demand, the massive harvesting of wild Huang-Qi has led to serious damage to the resources thereof, making it near the verge of extinction. Consequently, *A*. *membranaceus* var. *mongholicus* has been designated as endangered in the “China Rare Endangered Plant Directory” [14]. At present, commercial Huang-Qi is mainly cultivated. In 2017, Huang-Qi production areas in China were estimated at 173,000 ha, covering the entirety of northeast, northwest, and central China [12,15]. These widely different habitats have led to confusion in the Huang-Qi species, inconsistency in medicinal material quality, and complex population genetic diversity [16]. As a result, exploring the diversity of Huang-Qi germplasm resources will help to scientifically guide the commercialization of high-quality medicinal Huang-Qi, fundamentally addressing the contradiction between market demand and production quality.

With the development of socio-economics, the global consumption demand for Huang-Qi continues to grow and the demand for high-quality varieties is becoming increasingly urgent. Nonetheless, there are currently numerous challenges in Huang-Qi breeding, including insufficient understanding of germplasm resources, rudimentary breeding techniques, fragmentary breeding information, and low breeding efficiency, among other limitations. This paper provides a comprehensive and systematic overview of Huang-Qi germplasm resources and genetic breeding, focusing on the current status and diversity of germplasm resources, molecular marker-assisted breeding, breeding methods and their applications, and the prospects of molecular breeding. Its objective is to provide support for the literature and a theoretical basis for Huang-Qi breeding.

## 2. Germplasm Resources

### 2.1. Astragalus Species Resources and Morphological Classification

**Figure 1:**
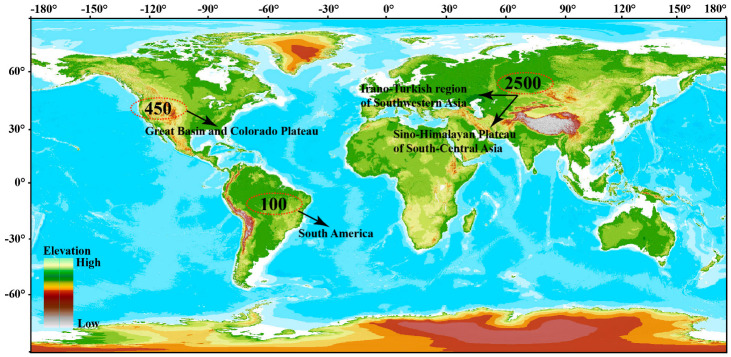
Major centers of distribution of *Astragalus* in the world.

*Astragalus* L. is a large genus of Fabaceae plants with about 2500–3000 species worldwide [17]. It predominantly inhabits the cold to warm arid and semiarid mountainous regions of the Northern Hemisphere and South America. The genus exhibits its greatest diversity in the southwest Asia region of Iran and Turkey (1000–1500 species), the Himalayan plateau of central and southern Asia in China (550 species), and the Great Basin and Colorado Plateau of western North America (450 species) [17] (Figure 1). However, Eurasia, especially the drier mountainous areas of southwestern and south-central Asia and the Himalayas, is considered the center of origin and diversity for *Astragalus*. According to statistical data from the literature and various flora as of September 2023, there are 401 species of *Astragalus* (221 endemic) in 59 sections (2 endemic) in China [18]. The plants of this genus are mainly distributed in the Qinghai–Tibet Plateau (QTP), Mongolian Plateau, and northwestern China, where it grows in dunes, wetlands, and grasslands at altitudes of 2000–6000 m. It is well-known that the uplift of the QTP at the end of the Tertiary period led to dramatic changes in the climate and topography of East Asia, which influenced the formation of a center of biodiversity there within the plant community [19]. In particular, *Phyllolobium* (41 species), a closely related genus derived from *Astragalus* in recent years, is mainly distributed in the QTP and adjacent areas of the Hengduan Mountains [19].

The study of the genus *Astragalus* originated in the early 18th century, with the botanist Tournefort having knowledge of such plants in 1700 and distinguishing between *Tragacantha* and *Astragaloides* [20]. In 1753, the botanist Linnaeus first described *Astragalus* in his “Species Plantanun”, which recorded 33 species of *Astragalus* and also described *Phaca* L. as a separate genus with three species [21]. In 1802, Candoll De classified *Astragalus* plants into three genera, *Astragalus*, *Phaca*, and *Oxytropis*, based on the characteristics of the keel petals and the number of ovary chambers and was the first to separate the genus *Oxytropis* from *Astragalus* [17].

As research progresses, studies of the subordinate classification within the genus *Astragalus* have become increasingly rich, focusing mainly on the two taxonomic levels of “subgenus” and “section”. In 1857, Kocb was the first to divide 19 species of *Astragalus* plants into three sections in his “Synopsis Florae Germanicae et Helveticae” [22]. In 1864, Gray divided the 115 species of *Astragalus* into 27 sections and merged *Phaca* L. into the genus *Astragalus* again [23]. In 1868, the botanist Bungesy systematically classified 971 species of *Astragalus*, dividing these taxa into 105 sections, and, for the first time, proposed the division of subgenera above the level of sections [24]. Simultaneously, *Phaca* L. was designated as Subg. *Phaca* (L) Bunge within *Astragalus*, and other classifications were divided into seven subgenera: Subg. *Pogonophace* Bunge, Subg. *Hypoglottis* Bunge, Subg. *Trimeniaeus* Bunge, Subg. *Tragacantha* Bung, Subg. *Calycophysa* Bunge, Subg. *Cercidothrix* Bunge, and Subg. *Calycocystis* Bunge (Table 1). In 1872, Boissier isolated a category of annual plants characterized by their hairy middles and established the subgenus Subg. *Epiglottis* [25]. In 1880, Bunge separated the taxa with distinctly leathery pods in the subgenus *Astragalus* as a new subgenus, Subg. *Caprinus*, from Subg. *Phaca*, known as Subg. *Astragalus* in “Flora of China” [18]. Therefore, the genus is divided into 10 subgenera (Table 1).

**Figure 2:**
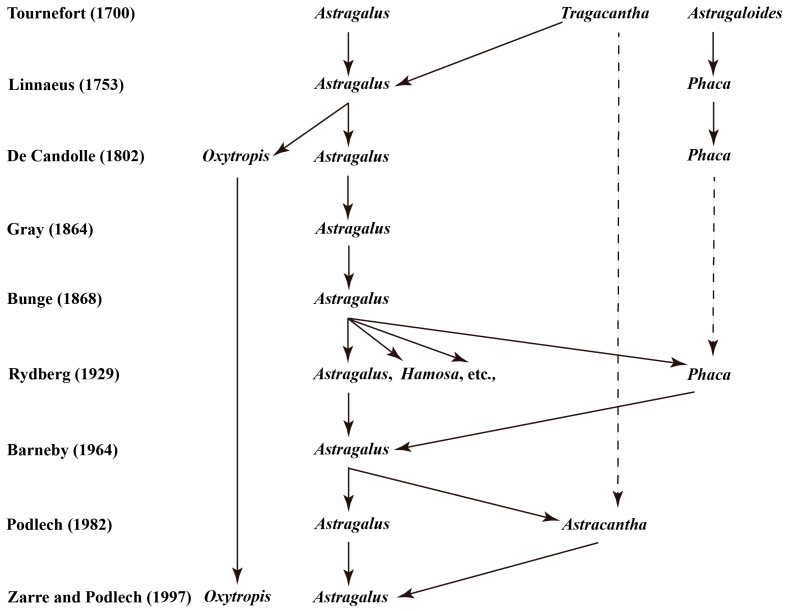
Major centers of distribution of *Astragalus* in the world (after Wojciechowski et al., 1999 [31] with some modifications).

However, there have always been different views on the classification of “subgenera”. In 1929, Rydberg classified 564 species of *Astragalus* into 28 genera, including *Astragalus*, *Phaca*, and *Hamosa*, and established 82 sections [26]. In 1964, Barneby classified the 554 North American species of *Astragalus* into *Phaca*, *Hypoglottis*, *Trimeniaeus*, *Cercidothrix*, *Homalobi*, *Piptolobi*, and *Orophaca*, with 93 sections [27]. In 1983, Podlech divided *Astragalus* into only two subgenera and elevated Subg. *Astracantha* to a new genus [28] (Table 1). In addition, the basal group of *Astragalus* has branched out into several smaller genera [29,30]. In 2013, Podlech and Zerra eliminated the subgenus classification for Old World *Astragalus* and used sections directly as a generic unit [18]. This practice has also been adopted by “Flora of China”. In summary, the systematic taxonomic research of *Astragalus* has undergone a long journey, and the classification of subordinate levels and species is still ongoing (Figure 2).

### 2.2. Geographic Distribution of Huang-Qi

**Figure 3:**
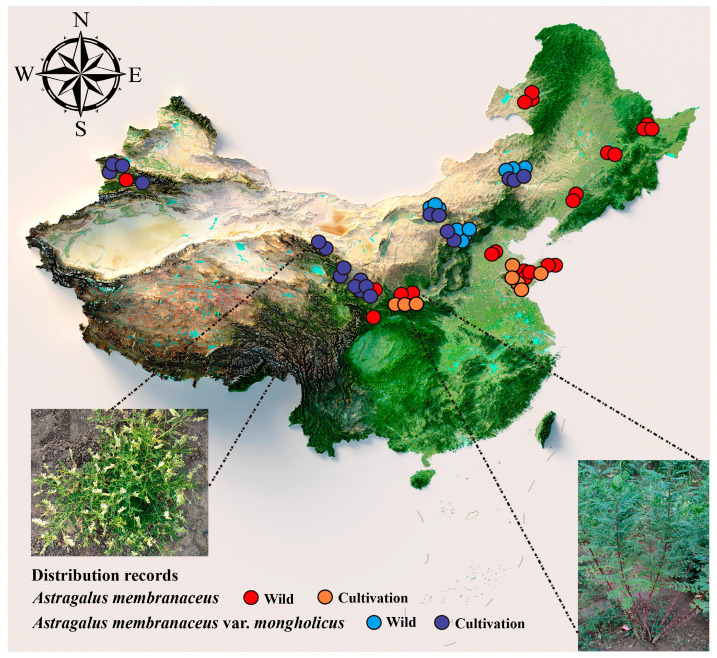
Geographical distribution of wild and cultivation of Huang-Qi. Dots on the map indicate the locations where wild and cultivated species of Huang-Qi were collected. Red dots indicate wild *A*. *membranaceus*, orange dots indicate cultivation *A*. *membranaceus*, blue dots indicate wild *A*. *membranaceus* var. *mongholicus*, and purple dots indicate cultivation *A*. *membranaceus* var. *mongholicus*.

According to the 2020 edition of the Chinese Pharmacopoeia, *A*. *membranaceus* and *A*. *membranaceus* var. *mongholicus* are the primary sources of Huang-Qi. The wild form of *A*. *membranaceus* is widely distributed across various provinces, including Heilongjiang, Jilin, Liaoning, Inner Mongolia, Hebei, Shanxi, Shaanxi, Ningxia, Gansu, Qinghai, Xinjiang, Shandong, and Sichuan. It primarily grows at the edges of forests or in sparse shrublands [32]. Artificially cultivated *A*. *membranaceus*, due to its well-developed roots, thick short primary roots, and abundant branches, is also known as “Ji Zhua-Qi” [33]. It is distributed in areas such as Wendeng City and Weifang City in the eastern Shandong Province and Xunyi County in Shaanxi Province and grows mainly in loamy soil zones at an altitude of 700–800 m [34]. The distribution area of wild *A*. *membranaceus* var. *mongholicus* is relatively narrow; it is mainly produced in Inner Mongolia, Hebei, and Shanxi, typically growing on sunny grassy or mountain slopes [12]. Currently, *A*. *membranaceus* var. *mongholicus* is mainly planted artificially and distributed in the Inner Mongolia, Shanxi, Gansu, Qinghai, and Hebei areas on half-slopes (Figure 3).

### 2.3. Status of Huang-Qi Resources

The resource supply of Huang-Qi has undergone changes from wild to cultivated, its distribution has transitioned from traditional to new production areas, and the scope of its application has expanded from traditional Chinese medicine clinical modulation to the raw materials of proprietary Chinese medicines and therapeutic food and health care [12]. Before the 1950s, Huang-Qi primarily relied on wild and semi-wild resources, which could not only satisfy the demand but also ensure the quality of the medicinal herbs, with *A*. *membranaceus* var. *mongholicus* being the leading variety [35]. The research of Zhan et al. revealed that the distribution area of Huang-Qi has gradually shifted from the southwest to the northeast over more than two thousand years of historical evolution [36]. Since the Qing Dynasty, Shanxi and Inner Mongolia have been regarded as high-quality producers of *A*. *membranaceus* var. *mongholicus*, while the three northeastern provinces are authentic producers of *A*. *membranaceus* [36]. In contemporary times, Huang-Qi produced in Shanxi, Shaanxi, Gansu, and Inner Mongolia is considered of the best quality [37].

In recent years, cultivated Huang-Qi has gradually dominated the medicinal herb market, leading to an urgent need for increased production and ensured quality [38]. Due to the scarcity of wild Huang-Qi resources, the area under artificial cultivation has expanded, highlighting the importance of expanding the scope of medicinal herbs and selecting and breeding superior germplasm. Currently, few Huang-Qi varieties have been selected and bred. The literature reports indicate that the Dry Farming Scientific Research Center in Dingxi, Gansu Province, used hybrid selection to develop new Huang-Qi lines 94-01 and 94-02, resulting in a yield increase of more than 15% compared to local farm varieties [39]. Additionally, hybrid selection produced the new variety 9118, characterized by a dwarfed plant structure, a long main root, fewer branches, increased resistance to root rot and powdery mildew, and a weight gain of over 20% [40]. Modern genomic and transcriptomic technologies offer new and rapid methods for identifying functional genes related to the synthesis of active ingredients and disease resistance in Huang-Qi. Transcriptomic analysis revealed that key genes involved in the biosynthesis of flavonoid components, such as PAL, 4CL, CCR, COMT, and DFR, were down-regulated during fruiting and up-regulated during fruit ripening in *A*. *membranaceus* var. *mongholicus*, which correlated with the trend of total flavonoid accumulation in the plant [41]. Chen et al. performed a genome-wide transcriptome analysis of *A*. *membranaceus* var. *mongholicus* to identify genes necessary for its metabolism and to analyze their expression patterns [42]. It was found that most genes involved in isoflavone biosynthesis had the lowest expression in leaves and the highest expression in stems. These studies provide a basis for further exploration of the biosynthetic mechanisms of important bioactive compounds in *A*. *membranaceus* var. *mongholicus*.

### 2.4. Huang-Qi Substitutes of Different Origins

**Figure 4:**
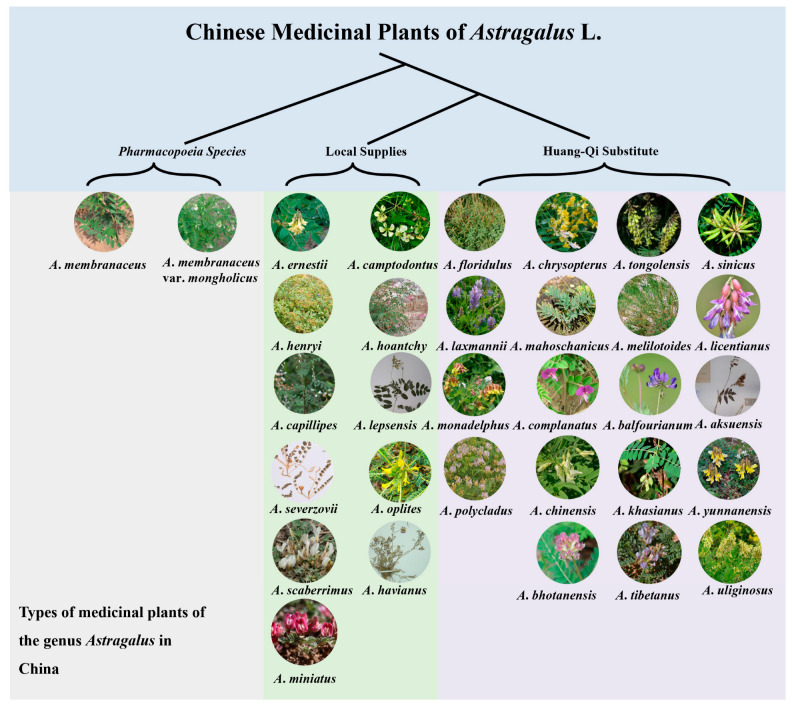
Introduction to the medicinal plant resources of *Astragalus* in China.

According to research reports, beyond the two Astragali radix species defined in the pharmacopeia, many regions use *Astragalus* or other genera of plants as substitutes for Astragali radix or as local standbys, mainly including plants from Subgen. *Phaca* Bunge, Subgen. *Astragalus*, Subgen. *Pogonphace* Bunge, Subgen. *Cercidothrin* Bunge, and Subgen. *Hypoglottis* Bunge [43] (Figure 4). Most of the medicinal parts are the roots, but there is some use of the whole plant or seeds as medicine (Table S1). The research of Qian et al. indicates that Yunnan is one of the distribution centers for *Astragalus* plants, with 13 species of local Astragali radix and 5 species of substitutes [44]. Gang et al., in their survey of the medicinal plant resources of the *Astragalus* genus in Qinghai Province, found 64 species of *Astragalus* there, with 7 extensively used by locals for medicinal purposes [45]. An investigation by Wang et al. revealed that in the main production areas of Astragali radix, besides *A*. *membranaceus* var. *mongholicus* and *A*. *membranaceus*, other species of the *Astragalus* genus are used as medicinal materials, including *Astragalus ernestii*, *Astragalus floridulus*, *Astragalus tongolensis*, *Astragalus chrysopterus*, and *Asfragalus monadelphus* [46] (Table S1). These are generally herbs that are customarily used in this region and are mostly self-produced and -marketed.

## 3. Genetic Diversity of Huang-Qi Germplasm Resources

The genetic diversity of germplasm resources is the basis for germplasm identification, genetic resource conservation, and breeding program design [47]. The study of the genetic traits of Huang-Qi is beneficial for dissecting the genetics of germplasm resources, exploring high-quality genes, developing new varieties, and promoting the sustainable utilization of Astragali radix germplasm resources.

### 3.1. Species Diversity

The genus *Astragalus* is a polymorphic group with numerous species and significant variation, with 2500–3000 species worldwide, primarily distributed in temperate regions of the Northern Hemisphere and South America and with widespread distribution also in central and western Asia [17]. The plant resources of *Astragalus* in China are abundant, and common species include *A*. *membranaceus* var. *mongholicus*, *A*. *membranaceus*, *Astragalus hoantchy*, *Astragalus henryi*, *Astragalus yunnanensis*, *Astragalus khasianus*, and *Astragalus laxmannii*, the first two of which have been included in the 2020 edition of the “Chinese Pharmacopoeia” [48]. Field investigations have revealed that the main growing areas of *A*. *membranaceus* var. *mongholicus* are located in Longxi, Tanchang, Weiyuan, and Zhangxian in Gansu and Lund in Ningxia. In recent years, production has steadily increased in the traditional growing regions of Shanxi and Inner Mongolia [48]. Shandong is the main cultivation area of *A*. *membranaceus*, which is also distributed in the northeast as well as in Zizhou and Xunyi in Shaanxi [48]. Wild *A*. *membranaceus* var. *mongholicus* has a wide distribution range, spanning the southwestern regions of Sichuan and Yunnan; the northwestern regions of Shanxi, Shaanxi, and Gansu; the northern regions of Hebei and Shandong; and the northeastern regions of Liaoning and Jilin [12]. Compared to *A*. *membranaceus*, wild *A*. *membranaceus* var. *mongholicus* has a narrower distribution area and grows mainly in the semiarid high-altitude areas of Shanxi and Inner Mongolia [48].

### 3.2. Morphological Diversity

**Figure 5:**
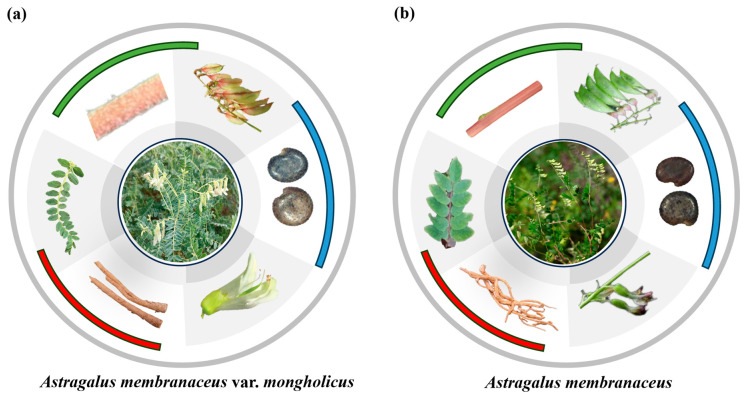
Phenotypic morphological differences between *A*. *membranaceus* and *A*. *membranaceus* var. *mongholicus*. Morphological analyses were used to differentiate between (**b**) *A*. *membranaceus* and (**a**) *A*. *membranaceus* var. *membranaceus* from six aspects: leaves, flowers, stems, pods, seeds and roots.

Morphology refers to the external phenotypic traits of a plant, which are the results of plant responses to changes in the growth environment [49]. Huang-Qi has a wide distribution area, and its morphological variability is large [50]. There are many variations and differences in leaf morphology, stem color, flower color, and fruit pod color morphology depending on the growth years, soil type, and ecological environment. Xie et al. found that the leaflets of *A*. *membranaceus* var. *mongholicus* have slightly blunt tips, with colors appearing to be a variety of lime green, green, gray–green, and black–green, through artificial cultivation [51]. The plant stems and vines showed a completely red variegation on the side exposed to solar radiation. In addition, the flag blossom of the butterfly-shaped flowers appeared in light yellow, light red, and dark red. Zhu et al. investigated and found a variant type with a lavender color at the top of the flag petal [52]. The color of the pods was mainly green, but some of the pods showed varying degrees of red. *A*. *membranaceus* is divided into two types: early flowering and late flowering [48]. Cao et al. compared the polysaccharide content and total saponins in the roots of five different varieties of *A*. *membranaceus*, and the experimental results showed that the wild early-flowering varieties had the highest polysaccharide content, the cultivated late-flowering varieties had significantly lower polysaccharide content than the other varieties, and the cultivated early-flowering varieties were in the middle [53]. However, the late-flowering type of *A*. *membranaceus* has the highest total saponin content and is a high-quality germplasm resource with high saponin content. The main differences between the *A*. *membranaceus* and *A*. *membranaceus* var. *mongholicus* distributed in China are shown in Figure 5 and Table 2.

### 3.3. Genetic Diversity of Cytological Markers

Cytological markers indicate variations in chromosome number, size, shape, orientation, position, and banding patterns, along with differences in euchromatin and heterochromatin distribution [59]. Chromosomes are carriers of genetic material, and changes in their number and structure lead to changes in the genetic material of crops, providing an important basis for research on intraspecific differences, interspecific phylogenetic relationships, and identification of progeny of hybrids [60]. Yan et al. karyotyped the chromosomes of *A*. *membranaceus* or *A*. *membranaceus* var. *mongholicus*, revealing that both are diploid with a chromosome number of 2n = 16 [61]. Lin et al. demonstrated that the karyotype of somatic cell chromosomes in *A*. *membranaceus* var. *mongholicus* is 2n = 16 = 2L + 4M_1_ + 6M_2_ + 4S, comprising eight pairs of chromosomes and classified as type 2B [62]. Qiao et al. observed mitotic activity in root tips of *A*. *membranaceus* or *A*. *membranaceus* var. *mongholicus* seeds by root tip chromosome preparation. The findings indicated that the chromosome karyotype formula of *A*. *membranaceus* was 2n = 2x = 18 = 4sm + 14m, classified as type 2B, whereas *A*. *membranaceus* var. *mongholicus* had a karyotype formula of 2n = 2x = 18 = 6sm + 12m, classified as type 2C [63]. Although cytological markers are not influenced by environmental factors, several challenges remain. First, preparing chromosome sections requires high technical skill, and the number of available markers is limited. Second, it is challenging to observe changes in individual genes within chromosomes. Additionally, relying on a single cytological method to study genetic diversity cannot accurately identify germplasm resources in different individuals of the same population or species with identical chromosomes.

### 3.4. Molecular Genetic Diversity

DNA molecular markers can be used not only to identify the genetic diversity and relationships of plant germplasm resources but also as an important tool for molecular-assisted breeding [64,65]. In recent years, a variety of molecular markers have been applied to identification and genetic diversity studies of Huang-Qi, which has rich genetic diversity in China [48,66,67,68]. Different populations within the same species show genetic differentiation, and the greater the distance between provenances, the lower the degree of similarity between populations [48]. Wang et al. selected 15 samples of cultivated Huang-Qi from four different provinces, a total of 85 allelic variant sites were amplified using random amplified polymorphic DNA (RAPD) with 12 primers, and the proportion of polymorphic sites was 78.16% [69]. The Nei genetic distance was calculated, and the genetic similarity of Huang-Qi was found to be higher among geographically close provinces. The relationship between wild and cultivated *A*. *membranaceus* was analyzed using RAPD technology, and the results showed that the average genetic distance between the two was 0.7026, indicating that they are distantly related. The genetic distances of 16 different sources of *A*. *membranaceus* were distributed between 0.059 and 0.608, which shows that their genetic diversity is relatively rich [70].

Amplified fragment length polymorphism (AFLP) molecular markers were used to study the genetic diversity of 17 cultivated *A*. *membranaceus* var. *mongholicus* populations and 5 wild *A*. *membranaceus* populations in the main Huang-Qi production areas [57]. Zhou et al. used 11 pairs of primers to amplify 85 polymorphic sites, and the results showed that the differences between the various geographic regions of *A*. *membranaceus* var. *mongholicus* were not obvious but the differences between those of *A*. *membranaceus* were very significant, which led to the inference that there were obvious differences between these two varieties [57]. ISSR molecular markers were applied to assess the genetic diversity of a total of 26 samples from 10 provinces, including Shanxi, Inner Mongolia, and Ningxia [71]. The results showed that the polymorphism ratio of Huang-Qi was 95.16% in Shanxi, followed by Inner Mongolia with a polymorphic allele variation rate of 92.02%, while Liaoning was at the lowest level with an allele variation rate of 43.19%. In addition, all genetic diversity indices were lower in the wild populations than in the cultivated populations, indicating that the level of genetic diversity in the former was lower than in the latter. He et al. used expressed sequence tag (EST) molecular marker technology to introduce the SSR of the soybean genome into an SSR locus study of Huang-Qi, which provided new research ideas for exploring the genetic diversity of Astragali radix [72].

In summary, since 2004, molecular markers at the DNA level have been applied to the analysis of molecular genetic diversity in Huang-Qi. However, each DNA molecular marker technique has certain limitations toward its application in the study of molecular genetic diversity in Huang-Qi to a certain extent. In addition, there have been few studies on the molecular genetic diversity of Huang-Qi in the last five years, and only the SSR molecular marker technique is common. Single-nucleotide polymorphism (SNP) markers have the advantages of genome-wide coverage, high throughput, and site specificity [73]. Currently, SNP markers have become attractive alternatives to SSR markers given the progress in genomic research and high-throughput sequencing.

## 4. Genetic Breeding

### 4.1. Genetic Research

Genetic diversity is an important variable for wild plant conservation and also a way to investigate the evolution of species in different habitats [74]. Uncovering the genetic mechanisms of species helps to understand the genetic diversity within populations and provides important guidance for breeding. In the actual production process, self-incompatibility, the mixing of field populations, and the confusion of germplasm resources have seriously restricted the genetic study of Huang-Qi [75]. However, in recent years, several scientific institutions have been initiated to focus on Huang-Qi, which has led to a gradual deepening of the understanding of its genetic basis.

### 4.2. Trait Inheritance

The transition of Huang-Qi from wild to cultivated began in the 1950s, and systematic breeding was mainly carried out after the 1990s [76]. Huang-Qi breeders often focus on the plant leaf morphology, flower shapes, fruit pod color, and compatibility of various hybrid combinations but pay little attention to the genetic mechanisms behind these phenotypic traits. Based on the phenotypic characteristics of the hybrid offspring and parents, long primary roots, few aboveground branches, large underground root systems, and disease resistance are dominant intrinsic traits in Huang-Qi breeding [77]. In many *Astragalus*, significant differences exist in the content of isoflavonoids and astragalosides, especially in *A*. *membranaceus* and *A*. *membranaceus* var. *mongholicus*, where the content of astragalosides I and IV are particularly abundant and can be stably inherited by subsequent generations [78]. This characteristic makes *A*. *membranaceus* var. *mongholicus* (Dingxi, China) and *A*. *membranaceus* var. *mongholicus* (Inner Mongolia) hybrids of Huang-Qi high yielding and high quality [77]. The new Huang-Qi variety 9118 is a dwarf plant with good herb quality, and its main components are calycosin-7-O-beta-D-glucoside; ononin; and astragaloside I, II, and III, which can be stably inherited by offspring through cross-breeding [79]. Tian et al. used *A*. *membranaceus* var. *mongholicus* “HQHY3” (♀) × *A*. *membranaceus* var. *mongholicus* “HQWT6” (♂) as hybrid parents, and irrespectively of orthogamy or reverse crossing, the seed set rate in the resulting hybrids exceeded 33%, demonstrating significant compatibility [80]. Chen et al. analyzed the SP2 generation of Huang-Qi populations, finding that the coefficients of variation for phenotypic traits and the genetic diversity index were 29.37 and 1.27, respectively [81]. It is necessary to consciously expand the number of hybrid offspring and comprehensively record their phenotypic characteristics.

### 4.3. Classical Cytogenetics and Molecular Markers

Chromosomes are the carriers of genes, control heredity and variation, and possess species-specific characteristics [63]. Studying the karyotype, morphology, and quantitative analysis of Huang-Qi somatic chromosomes is crucial for understanding its genetic mechanisms, phylogenetic relationships, and evolutionary processes and for identifying distant hybrids. Li et al. analyzed the karyotype of the chromosomes of A. membranaceus by conventional root tip squash method and showed that the chromosomes were of medium size, with a range of absolute length variability of 6.4–1.8 µm, no B-chromosome or aneuploidy phenomenon was observed, and the karyotype formula was 2n = 8m + 8sm (2SAT), which is in agreement with the previous report [82]. In A. membranaceus, among the eight pairs of chromosomes, the first, second, fourth, and eighth pairs were near the middle of the centromeres, and the remaining four pairs were in the middle of the centromeres. The eighth chromosome pair has a satellite on its short arm, which is extremely short, with the satellite being more than twice the length of the arm (length ratio 3.59), classifying this karyotype as 2B and relatively symmetrical, according to Stebbins*’* classification [82]. Lin et al.’s somatic karyotype analysis of A. membranaceus var. mongholicus showed that the number of somatic cell chromosomes was 16 [62]. In A. membranaceus var. mongholicus, the relative lengths of the eight chromosome pairs ranged from 5.8% to 19.3%. The longest chromosome measured 4.94 μm, while the shortest was 1.48 μm, yielding a ratio greater than two. The total width of the chromosomes was 4.22 μm, with chromosome 1 being the long chromosome, chromosomes 7 and 8 being the short chromosomes, chromosomes 2, 3, and 4 being the medium-length chromosomes, and chromosomes 5 and 6 being the medium-short chromosomes. In addition, the somatic chromosome karyotype of A. membranaceus var. mongholicus was 2n = 2x = 16 = 2L + 4M_1_ + 6M_2_ + 4S, classified as type 2B, following Stebbins*’* model of evolution from symmetrical to asymmetrical forms [62].

Huang-Qi is ecologically grown over an extremely wide range, and its germplasm resources are diverse, while there is a mixing of germplasm resources in cultivated Astragali radix. Therefore, relying solely on traditional morphological markers is insufficient to meet the demands of modern breeding. In contrast to traditional morphological indicators, molecular markers have the characteristics of good polymorphism and high stability and can avoid the influence of the external environment and other objective factors on the evaluation of germplasm resources [65,74]. The evaluation of genetic diversity using these markers is crucial for the effective combination of breeding materials and the improvement of crop traits [83]. At present, the primary molecular markers used in studying the genetic diversity of Huang-Qi germplasm include amplified fragment length polymorphisms (AFLPs), random amplified polymorphic DNA (RAPD), Simple Sequence Repeats (SSRs), Inter-Simple Sequence Repeats (ISSRs), Sequence-Related Amplified Polymorphisms (SRAPs), DNA barcoding, transcriptome sequencing (RNA-seq), single-nucleotide polymorphisms (SNPs), and Genotyping by Sequencing (GBS). Different molecular markers have achieved good results in verifying and identifying this diversity [69,84,85,86]. Zhou et al. used the AFLP technique to differentiate Huang-Qi from different regions of China and its two varieties [57]. Na et al. identified the genetic polymorphism of Huang-Qi produced in China and Korea through RAPD, providing a reference for identifying the production areas of Huang-Qi [66]. Liu et al. analyzed the genetic diversity and genetic structures of 380 Huang-Qi samples of different varieties and from 17 origins and successfully grouped them into different clusters [13]. Wang et al. investigated the genetic diversity and variation among 300 Huang-Qi samples from 15 populations, additionally analyzing the correlation between soil factors, meteorological factors, and the genetic diversity of the Huang-Qi, and tested the correlation between the genetic distance and geographical distance of the Huang-Qi [87]. The SSR is mainly used to assess the polymorphism of genetic material. Hou et al. showed that genetic variation in Huang-Qi mainly existed within populations, and the genus *Astragalus* could be distinguished when the primer similarity was 0.46, but it was not possible to specifically distinguish between *A*. *membranaceus* and *A*. *membranaceus* var. *mongholicus*, suggesting that Huang-Qi has rich genetic diversity [88]. Qian et al. used SRAP molecular marker technology to analyze the results, which showed that the genetic backgrounds of different populations of Huang-Qi had a high degree of similarity, but there were differences in the sub-spectral bands, which indicated that Huang-Qi had a high degree of genetic diversity, but the kinship could not be determined based on geographic location [89]. Sun et al. used ISSR markers to study the relationship between genetic diversity and drought resistance in Huang-Qi, showing that its genetic resources can be divided into three types, with significant genetic variation, and that ISSR genetic differentiation plays an important role in the genetic variation within these resources [90]. Comparative genomic analyses demonstrated the dynamic nature of the *A*. *membranaceus* chloroplast genomes, which showed the occurrence of numerous hypermutation loci, frequent gene losses, and fragment inversions [43,91]. The 80 germplasm resources were divided into two subgroups, with significant differences in genetic background between the two subgroups, but little correlation with the geographical origin of the Huang-Qi germplasm [92]. Chen et al. constructed the first chromosome-level genome of *A*. *membranaceus* var. *mongholicus*, systematically studying its evolutionary genome and providing an important basis for the molecular-assisted identification of Huang-Qi [81].

### 4.4. Biochemical Marker

Biochemical analysis utilizes protein characteristics, including isozymes and storage protein markers, to estimate genotypic differences through electrophoresis and to determine phylogenetic relationships based on enzyme profile similarities [59]. Bai et al. used esterase isozyme marker technology to reveal that Huang-Qi exhibits common characteristic bands, with varietal specificity [93]. Zhang et al. efficiently identified the seeds of A. membranaceus or *A. membranaceus* var. *mongholicus* using seed esterase isozyme polyacrylamide gel electrophoresis [54]. Xie et al. conducted a systematic analysis of esterase isozymes in seeds of *A. membranaceus* var. *mongholicus*, focusing on two traits (striped and unstriped), using vertical slab polyacrylamide gel electrophoresis (PAGE). The results indicated that the phenotypic and genetic enzyme profiles of *A. membranaceus* var. *mongholicus* were not completely consistent, suggesting that the maternal plants of these phenotypes experienced differentiation in the inheritance of subsequent generations’ traits [94]. In addition, cluster analysis revealed that some phenological and sub-phenological groups comprised seeds of the same phenotype. This observation may be linked to the significant morphological diversity within the heterogeneous group *A. membranaceus* var. *mongholicus*.

### 4.5. Traditional Breeding

The transition of Huang-Qi from wild to cultivated began in 1950 and was completed by 1984, spanning a total of 24 years [76]. During this stage, technological advancement was chiefly evident in the transition from direct seeding to the use of nurseries and transplantation and the improvement of planting methods from single vertical planting using manpower to flat-loaded planting that combines manpower and animal power, as well as the change from a basic understanding of the treatment of pests and diseases to the conducting of systematic research and the widespread dissemination of effective control techniques for these factors [76]. Meanwhile, 15 cultivated Huang-Qi varieties with a rich diversity of plant stem shape and shape as a whole have been developed through artificial introduction, domestication, selection, and breeding, and that number is still increasing. At present, systematic, cross-, and selective breeding are the most common methods of breeding Huang-Qi. In addition to these three methods, other techniques, such as radiation mutation, chemical, and space mutation breeding; tissue cultures; cell engineering; and gene cloning, have been used to breed new Huang-Qi cultivars [77]. The thicker seed coat of Huang-Qi seeds, particularly the early-maturing types that are firmer (iron seeds in Chinese), limits the ability of chemical mutagens and rays to penetrate. Huang-Qi has successfully established a regenerative plant system through tissue culture techniques; however, there are still difficulties in the mass production of seedlings for agricultural use using in vitro culture methods. These limitations significantly hinder the advancement of the aforementioned breeding techniques, resulting in their limited effectiveness [80,95].

### 4.6. Selective Breeding

Selective breeding is one of the most commonly used methods in Huang-Qi breeding, focusing mainly on the directional selection of plant height, leaf color, bud mutations, and stem color as a direct method to improve desirable traits [96]. Mixed selection involves selecting excellent individuals from existing varieties or materials based on objectives and breeding them to develop new cultivated varieties. In China, most new Huang-Qi cultivars were selected using this method before 2013. Regarding the selection and breeding of Huang-Qi variety resources, the overall progress has been slow, with few excellent-variety strains. Between 1994 and 2015, only Gansu successfully selected new cultivars of *A*. *membranaceus* var. *mongholicus* (LQ 1, LQ 2, LQ 3, and LQ 4) and Shandong selected and bred new cultivars of *A*. *membranaceus* (WH11) [77,97,98]. Although this breeding method is simple, sowing seeds and transplanting seedlings require much land, and the workload required to select excellent individuals is very heavy. With the shortage of land and increasing labor costs, this breeding method is gradually being replaced. In recent years, with the advancement of tissue culture technology, individuals exhibiting significant variations in plant height, leaf color, bud mutation, and stem color have been selected and utilized in asexual reproduction techniques to establish plant regeneration systems, thereby cultivating new varieties [80].

### 4.7. Molecular Breeding

Molecular breeding, as a method of genetic breeding at the molecular level, represents a combination of traditional breeding and genetic engineering techniques [99]. In addition, molecular breeding techniques are valued for their high precision, strong adaptability, and the rapid reduction in breeding cycles [100]. At present, the tissue culture of Huang-Qi mainly utilizes cotyledons, hypocotyls, stem segments, and the leaves of its sterile seedlings as explants [80]. By adjusting the concentration of exogenous hormones in the culture medium, suitable conditions for inducing the shape of healing tissue and regenerating plants have been screened and seedlings induced. The establishment of the regeneration plant system of Huang-Qi not only provides key technical support for the preservation of high-quality germplasm resources and the rapid breeding of seedlings, as well as their development and utilization, but also lays the foundation for carrying out genetic transformation and gene editing work in Huang-Qi. Despite the numerous challenges associated with the molecular breeding of Huang-Qi, the molecular mechanisms underlying various agriculturally and practically significant traits of Huang-Qi have been extensively studied. These molecular studies have laid a foundation for the future molecular breeding of Huang-Qi [101,102].

### 4.8. Flower and Leaf Color

Flowers are the reproductive organs of plants and are important for their survival [103]. Flower color is one of the important morphological characteristics of plants; because it is genetically stable and not easily affected by environmental factors, it can be used as an important genetic marker that plays a key role in field variety selection and cross-breeding [104,105]. Flower color depends mainly on the type and degree of accumulation of floral pigments, and different floral pigments result in differences in petal color [106]. The pigments in the petals of Huang-Qi are flavonoid compounds, including anthocyanins, flavones, and flavonols. The diversity of flower colors is mainly determined by three major groups of floral pigments, i.e., flavonoids, carotenoids, and other alkaloid-related, water-soluble pigments such as betaine and berberine, whose specific color patterns are regulated by the differential expression of their biosynthesis-related genes [107,108]. The metabolic pathways of flavonoids in Huang-Qi have been relatively well-resolved [109]. The biosynthesis of flavonoids is controlled by several structural genes, among which the key structural genes such as *PAL*, *C4H*, *4CL1*, *4CL2*, *CHS2*, and *CHI* are located upstream of the biosynthesis pathway, which has an important influence on flavonoid synthesis [110,111]. The structural genes *CHS*, *CHI*, *F3*′*H*, *DFR*, *ANS*, and *UFGT* play a crucial role in the synthesis of anthocyanins, among which *CHS*, *CHI*, and *F3*′*H* are the early genes of the anthocyanin synthesis pathway, *DFR* and *ANS* are the late genes of anthocyanin synthesis, and *UFGT* is the anthocyanidin transfer gene [112,113]. Changes in the expression levels of the *NHX* gene can cause flower color to change from red to purple and then to blue [114]. The expression levels of other structural genes, such as *PAL*, *4CL*, *CHS*, *F3*′*H*, *F3*′*5*′*H*, *DFR*, *UF3GT*, and *UF5GT*, also play a key role in determining the yellow and white flower colors [115].

## 5. Conclusions and Perspectives

### 5.1. Germplasm Diversity

Huang-Qi has a wide distribution area and a complex ecological environment, which result in morphological diversity and interspecific resource diversity. Genetic diversity is the basis of morphological diversity, but its mechanism in Huang-Qi remains unexplained. Future research should focus on the co-cultivation and off situ cultivation of different forms of Huang-Qi and combine them with genomics in order to gain a deeper understanding of its genetic mechanism, enrich genetic diversity, and lay the foundation for the selection and breeding of superior varieties. There are many species of *Astragalus* plants and abundant interspecific resources. Through visits to the market, this author discovered that there are cases where plants of the same genus, such as *Hedysarum polybotrys* (Hedysari radix, HongQi in Chinese), are passed off as medicinal Huang-Qi [116]. Therefore, alongside regulation of the authentic sourcing and sales processes of Huang-Qi, the related plants should be researched and developed in a rational manner. In addition, the medicinal value of the plants was determined by comparing the affinities between plants of the same genus and the basal species of Huang-Qi, as well as indicators such as the types and contents of the main active ingredients. Although large-scale cultivation has alleviated the shortage of wild resources of Huang-Qi to a certain extent, there is still a gap in the quality of cultivated products due to natural and artificial factors, and it is not conducive to the conservation of genetic diversity. Therefore, the introduction of wild varieties into the cultivation process contributes to the conservation of genetic diversity by promoting cultivation quality and yield through natural selection.

### 5.2. Breed Authenticity Protection

There are many production areas of Huang-Qi, including two newly formed ones (*A*. *membranaceus* var. *mongholicus* in Gansu and *A*. *membranaceus* in Shandong), and traditional ones co-exist in the resource pattern [12]. The authenticity of the Huang-Qi produced in the new and traditional production areas is a question that needs to be studied urgently. Local wild varieties have retained more of the authentic characteristics of Huang-Qi. Therefore, restoring wild Huang-Qi resources can help improve quality and authenticity and solve the problems of serious plant diseases, insect pests, and the insufficient accumulation of secondary metabolites in cultivated products. New production areas should establish germplasm resource libraries; strengthen the identification, evaluation, and selection of the quality of cultivated varieties; and introduce wild varieties with closer affinities to cultivated varieties in order to improve the authenticity of their products.

### 5.3. Breeding of Superior Seeds

Currently, there are few reports of the cultivation varieties of Huang-Qi, and the production of cultivars mainly relies on traditional breeding methods. However, with the development of modern molecular biotechnology, it is necessary to strengthen and modernize the molecular breeding research of this plant. The phenylalanine deaminase gene was found to regulate the phenylpropane metabolic pathway in medicinal plants in a gene cloning study of Huang-Qi [117]. Establishing a stable genetic transformation system for Huang-Qi as soon as possible is necessary to introduce functional genes for target characteristics into homozygous plants for directed cultivation. There are problems in the degradation of germplasm resources and mixed varieties in Huang-Qi cultivation. Parents with excellent specific traits should be selected to build a richer genetic population. QTL mapping or GWASs should be carried out in genetic populations using multicomponent and performance data to locate the genes and linkage markers associated with target traits. The application of validated markers for specific traits will expedite the breeding process. Recently, the first chromosome-level *A*. *membranaceus* var. *mongholicus* genome was published [118]. Its evolution was systematically investigated and key genes in the triterpene metabolism gene cluster were identified, which provided an important basis for the molecular-assisted identification of *A*. *membranaceus* var. *mongholicus* and further exploration of its molecular mechanisms underlying the chemical diversity of the active compounds.

## Figures and Tables

**Table 1:** Subgenera within *Astragalus*. Note: Bold taxon names possess aneuploid chromosome numbers in which Trimeniaeus phalanxes contains only one species *Astragalus wrightii* A. Gray (2*n* = 22) only distributed in North America (indicated by asterisks *). No subgeneric classification for South America is available.

	Bunge (1868/9)	Boissier (1872)	Bunge (1880)	Podlech (1983)	Zhang (2009)	New Worlds “Phalanxes” Barneby (1964)
Basifixed hair	*Phaca*	*Phaca*	*Phaca*	*Astragalus*	*Phaca*	Phacoid (6 sections, 13 species)
			*Caprinus*		*Caprinus*	
	*Calycophysa*	*Calycophysa*	*Calycophysa*		*Calycophysa*	
	*Hypoglottis*	*Hypoglottis*	*Hypoglottis*		*Hypoglottis*	Hypoglottis (1 section, 2 species)
	*Tragacantha*	*Tragacantha*	*Tragacantha*		*Tragacantha*	
	*Pogonophace*	*Pogonophace*	*Pogonophace*			
		*Trimeniaeus*	*Trimeniaeus*		*Trimeniaeus*	Trimeniaeus (1 section, 1 species)
Medifixed hair	*Cercidothrix*	*Cercidothrix*	*Cercidothrix*	*Cercidothrix*	*Cercidothrix*	Cercidothrix (2 sections, 4 species)
	*Calycocystis*	*Calycocystis*	*Calycocystis*		*Calycocystis*	
		*Epiglottis*	*Epiglottis*		*Epiglottis*	
Others	*Trimeniaeus* (basifixed hair and medifixed hair)			gen *Astracantha*	gen *Pogonophace*	Homoloboid * (46 sections, 194 species) Piptolobid * (35 sections, 192 species) Orophaca * (2 sections, 7 species)

**Table 2:** Characteristics of and differences in two medicinal Astragali radix distributed in China.

Plant Morphology	*Astragalus membranaceus*	*Astragalus membranaceus* var. *mongholicus*	Reference
Plant height	50–100 cm	40–80 cm	[54]
Stem	Upright stem and sparsely branched	Repent stem and much branched	[55]
Leaf	The number of leaves is small, generally 5–8 pairs, and the leaflets are long oval	The number of leaves is more, generally 9–11 pairs, and the leaflets are wide elliptic	[56]
Root	Most of them are chicken feet	Most of them are whiplash	[56]
Legume	There are bristles on the surface	The surface is setae-free	[57]
Seed	The seed umbilicus is heart-shaped with no obvious lines on the outer edge, and the germination pore is relatively wide	The seed umbilicus is oblong, the outer edge has obvious lines, and the germination pore is narrow	[48]
Flowering phase	July–August	May–June	[55]
Lavender flower variation	The variation rate is 50%	The variation rate is 59.3%	[58]

## Data Availability

All relevant data can be found within the manuscript.

## Supplementary Materials

The following supporting information can be downloaded at https://www.mdpi.com/article/10.3390/biology13080625/s1, Figure S1: PRISMA_2020_flow_diagram; Table S1: Current status of species, distribution and application of medicinal plants of the *Astragalus* in China.
