# Supplementary material for: Germplasm Resources and Genetic Breeding of Huang-Qi (Astragali Radix): A Systematic Review
Source: Biology (Basel). 2024 Aug 16;13(8):625. doi: 10.3390/biology13080625 (PMC11351161; doi:10.3390/biology13080625)
Supplement: Supplementary file 1 [file biology-13-00625-s001.zip › Supplementary/Figure S1.docx]

**Identification of studies via databases and registers**

Records removed *before screening*:

Duplicate records removed (n =2,518 )

Records marked as ineligible by automation tools (n = 1,197)

Records removed for other reasons (n = 3829)

Records identified from*:

Databases (n =49,913 )

Registers (n =24 )

**Identification**

Records screened

(n =42,369 )

Records excluded**

(n = 42,086)

Reports sought for retrieval

(n =283 )

Reports not retrieved

(n = 107)

**Screening**

Reports assessed for eligibility

(n = 176)

Reports excluded:

Reason 1 (n = 63)

Reason 2 (n = 47)

Reason 3 (n = 35)

etc.

Studies included in review

(n = 20)

Reports of included studies

(n = 11)

**Included**

Figure S1: PRISMA_2020_flow_diagram

*Consider, if feasible to do so, reporting the number of records identified from each database or register searched (rather than the total number across all databases/registers).

**If automation tools were used, indicate how many records were excluded by a human and how many were excluded by automation tools.

Source: Page MJ, et al. BMJ 2021;372:n71. doi: 10.1136/bmj.n71.

This work is licensed under CC BY 4.0. To view a copy of this license, visit <https://creativecommons.org/licenses/by/4.0/>
