# Supplementary material for: Germplasm Resources and Genetic Breeding of Huang-Qi (Astragali Radix): A Systematic Review
Source: Biology (Basel). 2024 Aug 16;13(8):625. doi: 10.3390/biology13080625 (PMC11351161; doi:10.3390/biology13080625)
Supplement: Supplementary file 1 [file biology-13-00625-s001.zip › Supplementary/Table S1.docx]

**Table S1** Current status of species, distribution and application of medicinal plants of the *Astragalus* in China

| **Species** | **Distribution** | **Medicinal part** | **Application status** | **Reference** |
| --- | --- | --- | --- | --- |
| *Astragalus hoantchy* | Inner Mongolia, Ningxia, Gansu, Qinghai and Xinjiang | Roots | Substitute for Astragali radix | Zhao et al., 2000 |
| *Astragalus camptodontus* | Sichuan and Yunnan | Roots | Yunnan substitute Astragali radix | Qian et al., 1997 |
| *Astragalus balfourianus* | Sichuan (Ganz, and Muli) and Yunnan | Roots | Yunnan substitute Astragali radix | Qian et al., 1997 |
| *Astragalus licentianus* | Gansu (Xiahe, and Minxian), Qinghai, Sichuan and Tibet | Roots |  | Gang et al., 1993 |
| *Astragalus complanatus* | Henan, Shaanxi, Ningxia, Gansu, Jiangsu and Sichuan | Seeds | The seeds are used in medicine | Qian et al., 1997 |
| *Astragalus henryi* | Shaanxi and Hubei | Roots | Substitute for Astragali radix | Zhao et al., 2000 |
| *Astragalus aksuensis* | Xinjiang | Roots |  | Zhao et al., 2000 |
| *Astragalus ernestii* | Sichuan, Yunnan and Tibet | Roots | Sichuan substitute Astragali radix | Wang et al., 1997 |
| *Astragalus monadelphus* | Gansu, Qinghai and Sichuan | Roots | Substitute for Astragali radix | Wang et al., 1997 |
| *Astragalus lepsensis* | Xinjiang | Roots | Xinjiang substitute Astragali radix | Zhao et al., 2000 |
| *Astragalus membranaceus* | Northeast, Northwest and North China | Roots | Astragali radix | Qian et al., 1997 |
| *Astragalus membranaceus*  var. *mongholicus* | Gansu, Heilongjiang, Inner Mongolia, Hebei, Shanxi, Shaanxi and Xinjiang | Roots | Astragali radix | Qian et al., 1997 |
| *Astragalus floridulus* | Gansu, Qinghai, Sichuan and Tibet | Roots | Sichuan substitute Astragali radix | Qian et al., 1997  Wang et al., 1997 |
| *Astragalus tongolensis* | Sichuan | Roots | Gansu, Qinghai and Sichuan substitute Astragali radix | Qian et al., 1997 |
| *Astragalus chrysopterus* | Sichuan, Hebei, Shanxi, Shaanxi, Gansu, Ningxia and Qinghai | Roots | Hebei, and Gansu substitute Astragali radix | Gang et al., 1993 |
| *Astragalus yunnanensis* | Sichuan, Yunnan and Tibet | Roots | Tibet substitute Astragali | Qian et al., 1997 |
| *Astragalus mahoschanicus* | Sichuan, Inner Mongolia, Gansu, Ningxia, Qinghai and Xinjiang. | Roots |  | Gang et al., 1993 |
| *Astragalus capillipes* | Inner Mongolia, Hebei, Shanxi and Shaanxi | Roots |  | Zhao et al., 2000 |
| *Astragalus melilotoides* | Provinces and districts north of the Yangtze River | Whole plant | Substitute for Astragali radix | Gang et al., 1993 |
| *Astragalus oplites* | Tibet and Xinjiang | Roots |  | Zhao et al., 2000 |
| *Astragalus severzovii* | Xinjiang | Roots |  | Zhao et al., 2000 |
| *Astragalus khasianus* | Yunnan and Tibet | Roots | Substitute for Astragali radix | Zhao et al., 2000 |
| *Astragalus chinensis* | Liaoning, Jilin, Heilongjiang, Inner Mongolia, Hebei and Shanxi | Seeds | The seeds are used in medicine | Zhao et al., 2000 |
| *Astragalus polycladus* | Sichuan, Yunnan, Tibet, Qinghai and Xinjiang | Roots |  | Zhao et al., 2000 |
| *Astragalus havianus* | Shaanxi | Roots |  | Zhao et al., 2000 |
| *Astragalus sinicus* | Provinces in the Yangtze River Basin | Whole plant, seeds | The seeds are used in medicine | Qian et al., 1997 |
| *Astragalus scaberrimus* | Northeast, Northwest and North China provinces | Roots |  | Zhao et al., 2000 |
| *Astragalus bhotanensis* | Guizhou, Sichuan, Tibet, Shaanxi and Gansu | Roots | Substitute for Astragali radix | Qian et al., 1997 |
| *Astragalus laxmannii* | Northeast, Northwest, and North China | Roots | Substitute for Astragali radix | Qian et al., 1997 |
| *Astragalus miniatus* | Inner Mongolia and Heilongjiang | Roots |  | Zhao et al., 2000 |
| Astragalus uliginosus | Inner Mongolia and north-eastern provinces | Roots | Substitute for Astragali radix | Zhao et al., 2000 |
| *Astragalus tibetanus* | *Xinjiang and south-west* | Roots | Yunnan and Xizang substitute for Astragali radix | Zhao et al., 2000 |

Qian, Z.G.; Jia, X.Y.; Dai, R.; Gu, D.S.; Pi, W.L. Studies on the species diversity of medicinal plants of Astragalus membranaceus in Yunnan Province, China. Journal of Yunnan College of Traditional Chinese Medicine. 1997,20 , 4.

Gang, J.; Guo, P.J. Medicinal resources of Astragalus spp. in Qinghai and its commercial herbs. Chin. Herb. Med. 1993, 16 , 15.

Wang, B.Q. Collation and quality study of commonly used Chinese herbal medicines varieties. Fujian Science and Technology Press. 1997

Zhao, M.; Duan, J. A.; Huang, W. Z. Current status and analysis of medicinal plant resources of Astragalus Linn. in China. Chin. Wild Pl. Resources. 2000, 6, 5-9.
